# F42. CHONDROTIN-6 SULFATE CLUSTERS: ASSOCIATION OF SYNAPTIC DOMAINS AND REGULATION OF SYNAPTIC PLASTICITY DURING FEAR LEARNING

**DOI:** 10.1093/schbul/sby017.573

**Published:** 2018-04-01

**Authors:** Gabriele Chelini, Cristina Berciu, Kanoelani Pilobello, Durning Peter, Jenkins Rachel, Moazzzam Kahn, Teniel Ramikie, Siva Subramanian, Kerry Ressler, Charalampos Pantazopoulos, Sabina Berretta

**Affiliations:** 1 Harvard Medical School McLean Hospital; 2 Oberlin College; 3 Lesley University

## Abstract

**Background:**

Emerging evidence from our group and others has brought the brain extracellular matrix (ECM) to the forefront of investigations on brain disorders. Our group has shown that organized perisynaptic ECM aggregates, i.e. perineuronal nets (PNNs) are decreased in several brain regions in people with schizophrenia (SZ) and bipolar disorder (BD). PNNs were detected by their expression of specific chondroitin sulfate proteoglycans (CSPGs), main components of the ECM, thought to play a key role in synaptic regulation during development and adulthood. Our studies have also shown that glial cells expressing CSPGs are altered in these disorders, suggesting a link between glial cell and PNN abnormalities. Finally, we have recently shown that novel CSPG structures, bearing a distinct CS-6 sulfation pattern and named CS-6 glial clusters, are decreased in the amygdala of people with SZ and BD. The morphology and function of CS-6 glial clusters is not currently known, but evidence from rodents and on the role of CSPGs in regulating synaptic functions strongly suggest that they may affect synaptic plasticity. We tested this hypothesis using a combination of human postmortem and rodent brain studies.

**Methods:**

High Resolution electron microscopy was used to investigate the ultrastructural organization of CS-6 glia clusters. A transgenic mouse model expressing green fluorescent protein in a subset of excitatory pyramidal neurons was used to investigate dendritic spines association with CS-6 glia clusters. Mice were exposed to a single session of auditory fear conditioning for a total of 15 minutes. Animals were euthanized 4 hours after behavioral test. Multiplex immunocytochemistry was used to visualize CS-6 clusters.

**Results:**

In human tissue, we show that CS-6 glia clusters are widespread in several brain regions, including the amygdala, entorhinal cortex, thalamus and hippocampus. Ultrastructural results show that CS-6 glia clusters are formed by CS-6 accumulations surrounding several dendrites. CS-6 expression was dected in astrocytes surrounding the dendrites, particularly in astrocytic endfeet enveloping dendritic spines, and within spines postsynaptic densities. Following auditory fear conditioning, marked changes of CS-6 glia clusters were observed in hippocampus regions dentate gyrus (g>1.5) and CA2 (g>1.5) and basolateral amygdala (g>1).

**Discussion:**

These findings suggest that CS-6 glia clusters may represent segregated microdomains, dynamically regulated during learning and contributing to the modulation of synaptic regulation machinery. Specifically, we postulate that astrocytes synthesize CS-6 CSPG and secrete it through their endfeet around dendrites, modulating structural plasticity of dendritic spines. These results suggest a relationship between the abnormalities in CSPGs expression and alteration in dendritic spines, two pathological landmarks observed in postmortem brains of people with SZ and BD.

